# TPEDCC-PEG-based photodynamic therapy exhibits potent therapeutic efficacy against echinococcosis by inducing oxidative stress and mitochondria-mediated apoptosis

**DOI:** 10.1371/journal.pntd.0013793

**Published:** 2025-12-19

**Authors:** Zhongxiu Ma, Luoyijun Xie, Li Wang, Zhi Wang, Hongqin Zhao, Lin Qiu, Zhichao Ma, Jiayu Tian, Leilei Shi, Miaomiao Yuan, Qi Xin

**Affiliations:** 1 Department of Pathogenic Biology, School of Basic Medical Sciences, Lanzhou University, Lanzhou, China; 2 Precision Research Center for Refractory Diseases in Shanghai General Hospital, Shanghai Jiao Tong University School of Medicine, Shanghai, China; University of Passo Fundo: Universidade de Passo Fundo, BRAZIL

## Abstract

Alveolar echinococcosis (AE) is a fatal zoonosis caused by the larval stage of *Echinococcus multilocularis*, but there are no fully effective treatments for this disease to date. The aim of this study was to investigate the potential therapeutic implication of photodynamic therapy (PDT) for *E. multilocularis*. We synthesized a novel nanophotosensitizer, TPEDCC-PEG, with aggregation-induced emission (AIE) properties and investigated its *in vitro* and *in vivo* PDT effects against the larval stage of *E. multilocularis*, as well as the anti-*Echinococcus* mechanism. TPEDCC-PEG exhibited excellent physicochemical properties and biocompatibility as a nanophotosensitizer. TPEDCC-PEG PDT, the treatment of TPEDCC-PEG with 410 nm laser irradiation, showed significant dose- and time- dependent protoscolicidal effect on *in vitro* cultured protoscoleces. Meanwhile, the infection experiments *in vivo* showed that the growth of protoscoleces survived after TPEDCC-PEG PDT was significantly suppressed *in vitro*, and the weight of metacestodes resulting from such protoscoleces in TPEDCC-PEG PDT mice was significant less than that in controls. Once every 3 days PDT administration (410 nm, 10 min) of TPEDCC-PEG (10 mg/kg) for 9 days in *E. multilocularis*-infected mice was highly effective in reducing the volume of metacestode cysts, resulting in a significant inhibition in metacestodes growth and severe structural destructions of metacestodes. Furthermore, TPEDCC-PEG PDT significantly increased the production of reactive oxygen species (ROS), inhibited the expression of glutathione in protoscoleces, and was accompanied by the depolarization of mitochondrial membrane potential. Western blot analysis revealed significantly reduced expression of the Bcl-2 protein alongside increased expression of caspase-3, Bax, and cytochrome c (Cyt C) proteins in protoscoleces following TPEDCC-PEG PDT, which hints the induction of apoptosis would associate with oxidative stress and involve the mitochondrial pathway. Taken together, the results suggest that PDT with TPEDCC-PEG, which exerted potent parasiticidal effects against *E. multilocularis* both *in vitro* and *in vivo*, could thus serve as a promising therapeutic option for echinococcosis.

## Author summary

Alveolar echinococcosis (AE) is a parasitic disease that poses a serious threat to human health. Due to the limitations of traditional treatments, the development of new, efficacious and safe therapeutic approaches is urgently needed. Photodynamic therapy (PDT) has been widely studied and applied cancers treatment owing to its advantages of target specificity, minimal invasiveness and low toxicity. Meanwhile, recent studies have found that PDT has a significant growth inhibitory effect on a variety of parasites, such as *Leishmania protozoa* and *Trypanosoma cruzi*, which provides an important theoretical basis for exploring the role of PDT in the treatment of AE. Therefore, in this study, we designed and synthesized TPEDCC-PEG, a photosensitizer with aggregation-induced emission properties, and investigated its PDT efficacy against *E. multilocularis*. TPEDCC-PEG PDT exerts significant anti-AE effects both *in vitro* and *in vivo* assay system. Furthermore, TPEDCC-PEG PDT can provoke the apoptosis of protoscoleces by oxidative stress and mitochondrial-mediated apoptotic pathways. These findings suggest that TPEDCC-PEG PDT can be developed as a promising method for AE therapy and may provide a new strategy for the treatment of human echinococcosis in the future.

## Introduction

Alveolar echinococcosis (AE) is a cosmopolitan zoonotic parasitic disease caused by the larval stage of tapeworm *Echinococcus multilocularis.* AE has a widespread distribution in the northern hemisphere, with the highest prevalence in Northern and Eastern Europe, North America, Japan, and North-western China [1]. Globally, a total of 0.3–0.5 million AE cases are diagnosed and AE causes approximately 18,000 (CIs 11,932–28,156) new cases every year, with a disease burden of 666,434 disability-adjusted life years (DALYs) [1]. Human AE is acquired through ingestion of food or water contaminated with tapeworm eggs which are released into the feces by the definitive hosts (mainly foxes and dogs), or by direct contact with infected animals. Upon infection, the larval stage (metacestode) of *E. multilocularis* proliferates and infiltrates the liver, thus developing into hepatic lesions (also known as “parasitic cancer”). Moreover, at a later stage of infection, the metacestode can metastasize to the lungs and brain, forming new lesions. Most AE patients suffer from abdominal pain, jaundice, and even liver failure. The mortality rate in untreated or inadequately treated AE patients is > 90% within 10–15 years of diagnosis [2,3]. As one of the world’s most lethal helminthic diseases, AE has been listed by the World Health Organization (WHO) as a neglected disease to be eradicated by 2050 [2].

Currently, the treatment of AE relies on surgery and is accompanied by anti-parasitic albendazole chemotherapy. Although surgical resection of hepatic parasitic lesions is the optimal treatment option, it is only recommended for patients with completely resectable lesions [4]. In advanced stages of AE, because the metacestodes proliferate and invade adjacent tissues or structures, the lesions cannot be removed radically by liver resection. This incomplete surgical resection therefore usually leads to local recurrence [5]. Considering the limitations of current therapy, treatment of AE remains a great challenge, and the development of novel therapeutic methods is necessary. As a result, in recent years, multiple *in vitro* and *in vivo* studies have investigated different modalities for the treatment of AE, including radiofrequency ablation [6], microwave ablation [7,8], high intensity focused ultrasound [9,10], ionizing radiation [11–15], and nanosecond pulsed electric fields [16]. However, these modalities have their own disadvantages, such as no clear-cut parasiticidal effects, limited *in vivo* efficacy, adverse side effects during treatment, and hospital technical limitations [13–15,17,18], leading to restrictions in their clinical application for AE therapy. Thus, investigate new, more effective, safer, and feasible therapies for AE is crucial.

**Fig 1:**
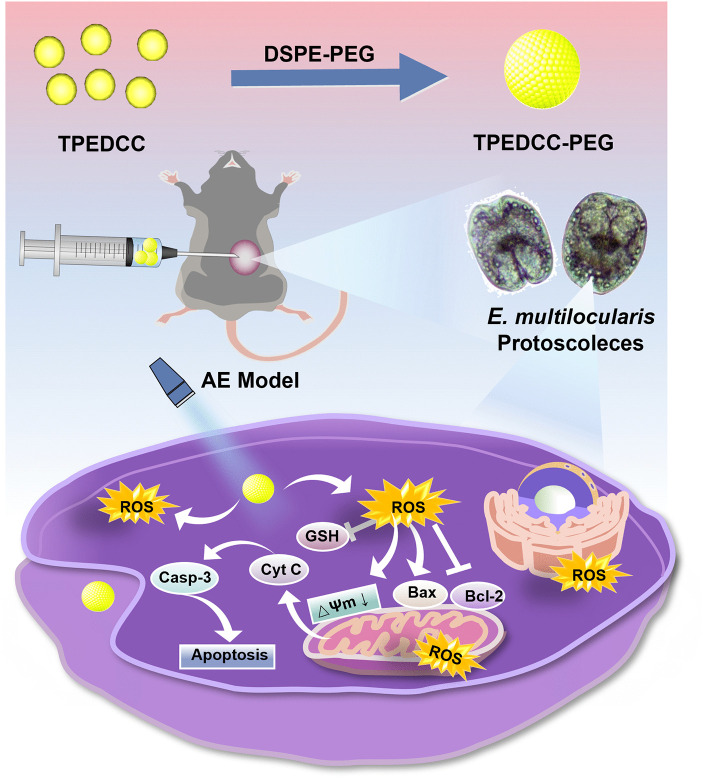
Schematic representation of the nanophotosensitizer TPEDCC-PEG exerts effect against *E. multilocularis in vitro* and *in vivo* through oxidative stress and mitochondria mediated apoptosis.

Photodynamic therapy (PDT), a physical therapy technique, exhibits spatiotemporal selectivity and therapeutic efficiency without causing systemic effects, making it a widely accepted clinical treatment for malignant tumors and other diseases [19–21]. PDT utilizes reactive oxygen species (ROS) generated by the local activation of photosensitizers (PSs) in the lesion sites under laser irradiation, ultimately eliminating the nidus [22–26]. In the organs of AE patients, *E. multilocularis* metacestodes exhibit some important similarities with malignant tumors [27], such as potentially unlimited proliferative capacity, infiltrative growth [28,29], and formation of metastases [30,31]. Therefore, we hypothesized that PDT may be an effective and alternative therapeutic treatment for AE. Herein, for the first time, we investigated the parasiticidal effects of PDT using a novel synthetic nanophotosensitizer TPEDCC-PEG on *E. multilocularis* metacestodes *in vitro* and *in vivo*. The results demonstrated that TPEDCC-PEG could exert a potent effect against the activity of *E. multilocularis* through mitochondria mediated apoptosis, indicating PDT with TPEDCC-PEG is a valuable and promising strategy for AE therapy (Fig 1).

## Methods and materials

### Ethics statement

All animal experimental procedures and animal management were conducted in compliance with the protocols approved by the Institutional Animal Care and Use Committee of Lanzhou University (jcyxy20210308). The mice were housed under laboratory conditions, with a 12-hour light/dark cycle and a controlled temperature, and provided with free access to water and commercial mouse chow throughout the study.

### Materials and reagents

DSPE-PEG and Tetrahydrofuran (THF) were purchased from the Macklin (Shanghai, China). The culture medium was purchased from Hyclone (Logan, UT, USA). The cell counting Kit-8 (CCK-8) (Cat# GC614) was purchased from Dojindo Molecular Technologies (Japan). The Alkaline phosphatase (ALP) (Cat# P0321S), ROS (Cat# S0033S) and Glutathione (GSH) (Cat# S0052) were obtained from Beyotime Biotechnology (Jiangsu, China). The terminal deoxynucleotidyl transferase-mediated dUTP nick end labeling (TUNEL) detection kits (Cat# 40306ES60) were obtained from Yeasen Biotechnology (Shanghai, China). Mitochondrial Membrane Potential Assay Kit with JC-1 (Cat# M8650) was purchased from Solarbio (Beijing, China). Cyt C (Cat# ab13575) was obtained from Abcam (Shanghai, China). Cleaved-caspase 3 (Cat# YM3431), Bcl-2 (Cat# YM3041), Bax (Cat# YM3619) and GAPDH (Cat# YM3029) antibodies were obtained from Imunnoway (Plano, USA).

### Synthesis and characterization of AIE PS TPEDCC-PEG

The 500 μL of TPEDCC (2 mg/mL in THF) [32] and DSPE-PEG (4 mg/mL in THF) were added to deionized water (10 mL). The mixture was then subjected to sonication for 1 h and stirred overnight (12 h). After that, the THF was evaporated at room temperature for 24 h in the dark. The final stock solution was stored at 4°C in the dark for further characterization and application. The morphology of AIE PS TPEDCC-PEG was observed with transmission electron microscopy (TEM) (JEM2010F, JEOL, Japan) operated at 200 kV. The size distribution and zeta potential were investigated by dynamic light scattering (DLS, NanoBrook). The UV-Vis spectrum was measured using a Multiscan spectrum (Multiskan Sky, Thermo Scientific, MA).

### Cytotoxicity of the AIE PS TPEDCC-PEG

Human Normal Liver LO2, Human Dermal Fibroblasts (HDF), and Mouse Fibroblast Cell L929 cell lines were purchased from Cell Resource Center, Chinese Academy of Science. Cells were seeded into 96-well plates at a density of 5,000 cells per well, incubated in RPMI 1640 medium containing 10% fetal bovine serum (FBS) and 1% penicillin-streptomycin for 24 h at 37˚C in a 5% CO_2_ atmosphere and then incubated with AIE PS TPEDCC-PEG at different concentrations (0, 2, 3, 6, 13, 25, 50, and 100 μg/mL) for 48 h. The cell viability was examined by the standard CCK-8 Kit.

### *E. multilocularis* protoscoleces preparation

The *E. multilocularis* strain, which was isolated from Xinjiang in China, was used in this study [33]. In short, *E. multilocularis* metacestodes were isolated aseptically from Mongolian gerbils (*Meriones unguiculatus*, obtained from Hangzhou Medical College, Zhejiang province, China) that had been intraperitoneally inoculated with 2,000 protoscoleces for 2 months. The metacestodes were cut into thin slices and strained through a metal tea strainer. The collected filtrate was filtered using a 70 mesh cell sieves and then strained through a 40-μm cell strainer for the removal of calcium particles. The collected protoscoleces were washed six times with sterile phosphate-buffered saline (PBS) and then cultured in RPMI 1640 culture medium without phenol red (12 mM HEPES, 2 mM glutamine, 100 U/mL penicillin, 100 μg/mL streptomycin) at 37°C and 5% CO_2_ conditions. The vitality of protoscoleces was assessed after staining the protoscoleces with 0.04% (w/v) trypan blue for 3 min. In our study, the initial vitality of protoscoleces was over 98%.

### *In vitro* TPEDCC-PEG PDT against *E. multilocularis* protoscoleces

To investigate the effects of TPEDCC-PEG PDT on *E. multilocularis* protoscoleces, the protoscoleces were cultured in 48-well tissue culture plates containing 300 protoscoleces per well in 500 μL of RPMI 1640 culture medium without phenol red at 37°C and 5% CO_2_ conditions. Then, different final concentrations (6.25, 12.5, 25, 50, 100, 150, 200, and 250 μg/mL) of TPEDCC-PEG were added into medium. The protoscoleces were incubated with TPEDCC-PEG for 2 h and then were exposed to a 410 nm laser for 10 min and imaged with a fluorescent microscope (NIKON ECLIPSE Ti2-E, Japan). The morphological changes of the protoscoleces were observed using an optical microscope and the viability was assessed by trypan blue staining test. The culture medium from all groups was harvested for examining *E. multilocularis* alkaline phosphatase (*Em*ALP) activity. Each treatment concentration was performed in duplicate, and the experiments were repeated twice. Additionally, protoscoleces were fixed in 2.5% glutaraldehyde in 100 mM sodium cacodylate buffer (pH 7.2) for the Scanning Electron Microscope (SEM) investigation. The samples were observed and imaged with a Hitachi S-450 SEM. To further confirm the protoscolicidal effect of TPEDCC-PEG PDT on protoscoleces, specific pathogen-free (SPF) BALB/c mice (female, 6–8 weeks, obtained from Lanzhou Veterinary Research Institute, Gansu, China) were intraperitoneally infected with 2,000 protoscoleces that had been pre-photodynamic treated *in vitro* with 12.5 μg/mL or 150 μg/mL TPEDCC-PEG, or a control substance. Ten weeks after the infection, all mice were humanely euthanized by inhalation of CO_2_ followed by cervical dislocation. The entire parasite tissue was isolated from the peritoneal cavity of the mice and placed in a disposable sterile plastic culture dish. Then, the wet weight of the parasite was determined with a precision electronic balance.

### Detection of ROS and GSH levels and mitochondrial function

The protoscoleces were pre-cultured in 48-well tissue culture plates, and divided into the following six groups: control group, laser group, 12.5 μg/mL of TPEDCC-PEG + laser group, 50 μg/mL of TPEDCC-PEG + laser group, 150 μg/mL of TPEDCC-PEG + laser group, and 250 μg/mL of TPEDCC-PEG + laser group. The protoscoleces were exposed to a 410 nm laser for 10 min. After treatment, the protoscoleces were incubated with 20 μM 2’,7’- dichlorodihydrofluorescein diacetate (DCFH-DA, Ex: 488 nm; Em: 525 nm) for 30 min to evaluate cellular ROS generation and imaged by a fluorescence microscope (NIKON ECLIPSE Ti2-E, Japan).

For GSH detection study, the protoscoleces were collected and washed twice with precooled PBS. Then, 200 μL proteinase removing reagent S solution was added. After sonication, the supernatant was collected by centrifuging at 10,000 rpm for 10 min and the GSH concentration was determined according to the manufacturer’s protocol.

For the evaluation of mitochondrial potential, the protoscoleces were collected and 500 μL of green fluorescent JC-1 was added. After incubating at 37°C for 20 min, the protoscoleces were washed three times with pre-cooled JC-1 staining buffer and then observed by a fluorescence microscope (NIKON ECLIPSE Ti2-E, Japan).

### Western blotting analysis

Western blotting was used to detect the protein expression in different groups. The protoscoleces were lysed on ice with RIPA lysis buffer (Solarbio, Beijing, China). After sonication, the lysates were collected by centrifuging at 4°C and 12,000 rpm for 10 min and protein concentration was measured by a BCA assay kit (Solarbio, Beijing, China). The protein samples were subjected to sodium dodecyl sulfate-polyacrylamide gel electrophoresis (SDS-PAGE) and transferred to a polyvinylidene fluoride membrane. The membranes were blocked with 5% nonfat milk at room temperature for 2 h, and then subjected to immunoblot analysis with primary antibodies against Bax (1:1,000), Bcl-2 (1:1,000), cleaved-caspase 3 (1:1,000), Cyt C (1:1,000), and GAPDH (1:1,000) at 4°C overnight. After washing with TBST buffer, the membranes were incubated with secondary antibodies (1:5,000) at room temperature for 1.5 h. Finally, the bands were detected using an enhanced chemiluminescence kit (Sunivew, Shenzhen, China).

### *In vivo* TPEDCC-PEG PDT performed in mice infected with *E. multilocularis* metacestode

SPF BALB/c mice (female, 6–8 weeks, obtained from Lanzhou Veterinary Research Institute, Gansu, China) were infected by subcutaneous inoculation [34] with 2,000 *E. multilocularis* protoscoleces into the flanks. After a month post-infection, the metacestode cysts with a dimension of approximately 150 mm^3^ developed and then the treatment was carried out. The metacestode cysts-bearing mice were randomly divided into four groups (n = 7 per group): control group, PBS + laser irradiation group, TPEDCC-PEG group, and TPEDCC-PEG + laser irradiation group. 50 μL of 4.5 mg/mL TPEDCC-PEG (10 mg/kg body weight for each mouse) or PBS were injected into the metacestode region of the mice. The laser groups were then treated with laser irradiation (410 nm) for 10 min once every 3 days for a period of 9 days. During the therapy, the length and width of the cysts, as well as the body weight of the mice were recorded before every injection. The cyst volume was calculated by this formula: V(mm^3^) = 1/2 **×** length (mm) **×** width (mm)^2^. At the end of the study, all mice were humanely euthanized by inhalation of CO_2_ followed by cervical dislocation, and death was confirmed by the absence of a heartbeat, respiration, and corneal reflex prior to necropsy. The cyst inhibition rate (CIR) was calculated using the following formula: CIR (%) = 100% × (mean cyst volume in each group before treatment - mean cyst volume in the group after treatment)/ mean cyst volume in each group before treatment. Whole blood via ocular sinus was collected from mice. Serums were separated by centrifugation (3,500 rpm, 10 min) at 4°C for blood biochemical analysis. The metacestode cysts were carefully isolated from the subcutaneous region of the mice. The heart, liver, spleen, and kidney of the mice, as well as ioslated metacestode cysts, were fixed in 4% formaldehyde and stained with hematoxylin-eosin (H&E) for histopathological examination.

### Assay for apoptosis in metacestodes of *E. multilocularis*

Apoptotic cells within the cysts were detected via the TUNEL assay using a commercial kit. Briefly, formaldehyde-fixed, paraffin-embedded metacestode cysts from the mice in the efficacy study were sectioned into 4-μm slices. Following deparaffinization in xylene and ethanol gradient dehydration, sections were digested with 20 μg/mL proteinase K (25°C, 20 min), rinsed thrice with PBS, and equilibrated with buffer (25°C, 20 min). TdT-mediated labeling was performed by incubating sections with FITC-12-dUTP and recombinant TdT enzyme (37°C, 60 min). Post-labeling washes utilized PBS containing 0.1% Triton X-100 and 5 mg/mL BSA. Nuclei were counterstained with 2 μg/mL DAPI (25°C, 5 min). Negative controls omitted TdT enzyme in the labeling solution. Fluorescent images were acquired using a microscope (NIKON ECLIPSE Ti2-E, Japan) under standardized exposure.

### Blood biochemical parameters assay

The serum levels of total protein (TP), albumin (ALB), total bilirubin (TBIL), direct bilirubin (DBIL), alanine aminotransferase (ALT), aspartate aminotransferase (AST), alkaline phosphatase (ALP), urea nitrogen (BUN), creatinine (CREA), and creatine kinase isoenzyme MB fraction (CKMB) were measured on the ROCHE cobas 8000 biochemical analyser (ROCHE, Basel, Switzerland).

### Statistical analysis

Statistical analysis was performed with GraphPad Prism 9.0.0 software. All experimental data are shown as mean ± standard deviation (SD). Multiple comparisons between more than two groups were analyzed using one-way ANOVA or two-way ANOVA parametric test. The results were considered statistically significant at *p *< 0.05.

## Results

### Characterization of AIE PS TPEDCC-PEG

**Fig 2:**
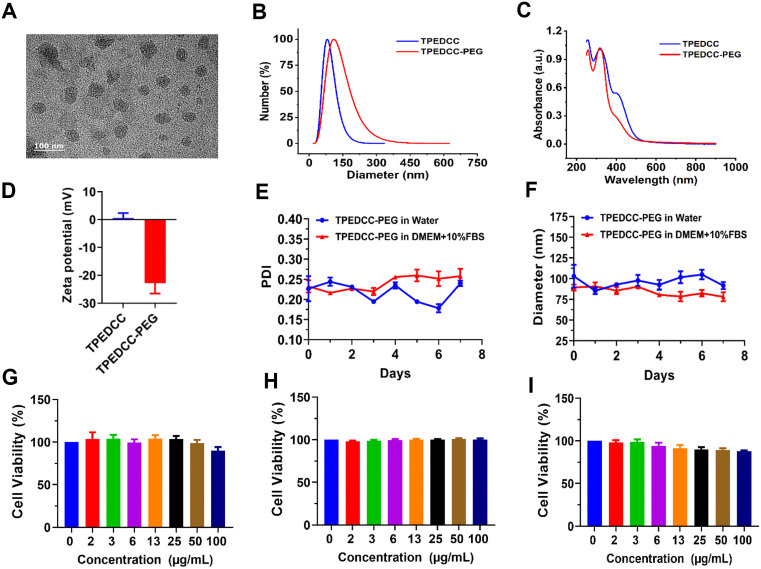
Characterization of AIE PS TPEDCC-PEG. (A) TEM image of TPEDCC-PEG, the scale bar is 100 nm. (B) Size distribution analysis of TPEDCC-PEG. (C) UV-Vis absorption spectra of TPEDCC-PEG. (D) Zeta potential distribution of TPEDCC-PEG. Analysis of particle distribution stability (E) and size stability (F) of TPEDCC-PEG in water and DMEM medium + 10% FBS, respectively. The cell viability of Human Normal Liver LO2 cells (G), Human Dermal Fibroblasts (H), and Mouse Fibroblast L929 cells (I) after incubation with TPEDCC-PEG at different concentrations for 48 h.

The detailed synthesis procedure of TPEDCC nanoparticles was discussed in our previous publications [32]. The AIE PS TPEDCC-PEG was obtained via the hybrid reaction between TPEDCC and DSPE-PEG. Then, we characterized the properties of TPEDCC-PEG. The TEM image illustrated that TPEDCC-PEG exhibits a uniform spherical morphology (Fig 2A). Then, DLS analysis confirmed that TPEDCC-PEG exhibits a diameter of 117.02 ± 8.12 nm (Fig 2B). The investigation of the optical properties showed that TPEDCC-PEG remained largely unchanged compared to the optical properties of TPEDCC, with the UV absorption peak observed at 350 nm. It also fluoresces red under 410 nm laser irradiation (Fig 2C). Furthermore, the zeta potential of TPEDCC-PEG was measured to be -22.73 ± 3.39 mV (Fig 2D). To further characterize the TPEDCC-PEG, the stability of TPEDCC-PEG was investigated by DLS in aqueous solutions and DMEM medium containing 10% FBS at different time points, respectively. As shown in Fig 2E and 2F, the TPEDCC-PEG was stable, whether in aqueous solutions or in DMEM medium + 10% FBS, with relatively consistent PDI and particle size for 7 days (Fig 2E and 2F).

For assessment of the biocompatibility of TPEDCC-PEG, Human Normal Liver LO2 cells, Human Dermal Fibroblasts, and Mouse Fibroblast L929 cells were employed to evaluate the *in vitro* cytotoxicity of the TPEDCC-PEG. As shown in Fig 2G–2I, the relative cell viability is higher than 90% at any tested concentration (2, 3, 6, 13, 25, 50, and 100 μg/mL), indicating negligible toxicity to all tested cell lines.

### PDT of TPEDCC-PEG exhibits *in vitro* efficacy against *E. multilocularis* protoscoleces

**Fig 3:**
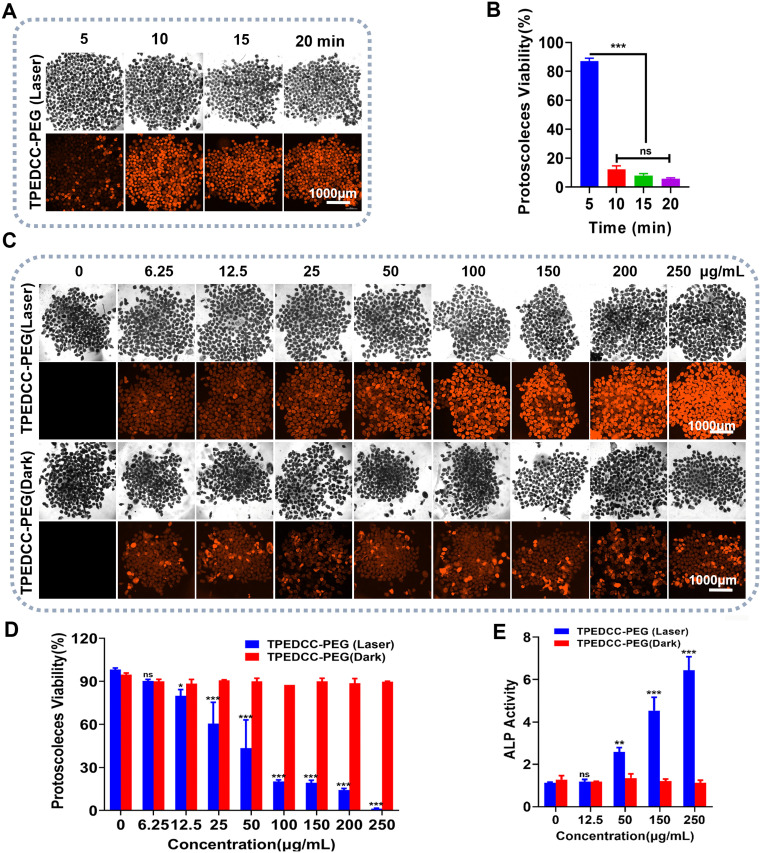
*In vitro* TPEDCC-PEG PDT against *E. multilocularis* protoscoleces. Fluorescence images (A) and the viability (B) of protoscoleces incubated with TPEDCC-PEG after being treated with laser irradiation for 5, 10, 15, and 20 min, respectively. ***p < 0.001. Fluorescence images (C) and viability (D) of protoscoleces incubated with TPEDCC-PEG at different concentrations without or with laser irradiation (410 nm, 10 min). The scale bar is 1000 μm. (E) *E. multilocularis* alkaline phosphatase (EmALP) activity in the medium supernatant after protoscoleces incubated with TPEDCC-PEG at different concentrations without or with laser irradiation (410 nm, 10 min). *p < 0.05*,* **p < 0.01, ***p < 0.001 vs. TPEDCC-PEG (Dark).

To investigate the PDT performance of TPEDCC-PEG against *E. multilocularis*, a TPEDCC-PEG PDT study was conducted both *in vitro* and *in vivo*. The *in vitro* PDT performance against *E. multilocularis* protoscoleces was assessed in response to a series of laser exposure times and concentrations of TPEDCC-PEG. As shown in Fig 3A and 3B, after irradiating for 5, 10, 15, and 20 min, the viability of protoscoleces decreased over time, presenting a time-dependent effect. However, although 15 and 20 min of laser irradiation seemed more efficient than 10 min, the difference in viability of protoscoleces did not show statistical significance. When incubated with a series of TPEDCC-PEG concentrations, without irradiation, the TPEDCC-PEG exhibited no toxicity to protoscoleces. In contrast, after irradiating for 10 min, the protoscoleces viability exhibited a negative correlation with the concentration of TPEDCC-PEG (Fig 3C and 3D). The survival rate of protoscoleces was less than 20% with 150 μg/mL and 200 μg/mL TPEDCC-PEG PDT treatment. Notably, almost all protoscoleces were killed after treatment with 250 μg/mL TPEDCC-PEG. The release of alkaline phosphatase (ALP) activity into the culture medium was determined as an indicator for the damages in destroying protoscoleces [35]. The results demonstrate that TPEDCC-PEG could result in an increased release of ALP activity in medium supernatant, meanwhile, this ALP release effect induced by TPEDCC-PEG PDT was dose-dependent (Fig 3E).

**Fig 4:**
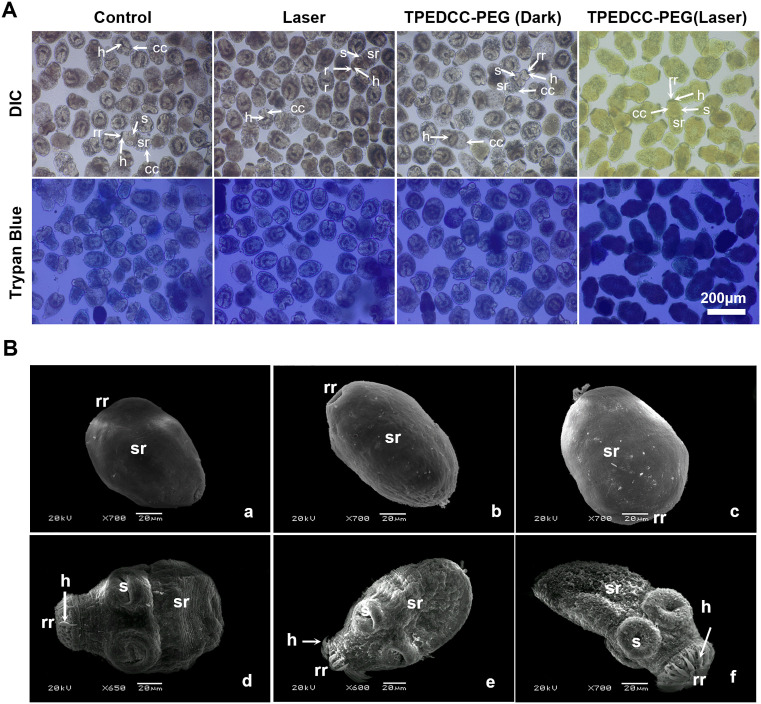
The effects of PDT treatment with TPEDCC-PEG on the morphology and integrity of protoscoleces. (A) Light microscopy of protoscoleces incubated with TPEDCC-PEG (150 μg/mL) upon laser irradiation (410 nm, 10 min). The scale bar is 200 μm. (B) The ultrastructure of protoscoleces by SEM analysis. (a) Control group, invaginated protoscoleces. (b) Laser group (410 nm, 10 min). Protoscoleces irradiated by laser (410 nm, 10 min) after incubation with 12.5 μg/mL (c), 50 μg/mL (d), 150 μg/mL (e), and 250 μg/mL (f) TPEDCC-PEG, respectively. The scale bar is 20 μm. rr, rostelar region; sr, soma region; s, suckers; h, hooks; cc, calcareous corpuscles.

Fig 4A shows the morphological changes of protoscoleces after PDT treatment of TPEDCC-PEG by optical microscope. The control protoscoleces exhibited normal structure, with invaginated form and intact hooks and calcareous corpuscles. The protoscoleces both in laser group and TPEDCC-PEG (Dark) group did not undergo obvious changes as compared to control protoscoleces. When protoscoleces incubated with TPEDCC-PEG (150 μg/mL) were irradiated with 410 nm laser for 10 min, almost all the protoscoleces showed marked morphological alterations, with evaginated form, cocked hooks, and reduced calcareus corpuscles, which coincide with the results of trypan blue staining. Furthermore, the TPEDCC-PEG PDT treatment resulted in dramatic morphology changes (Fig 4B) observed by SEM, which are in good agreement with the ALP activity results. After irradiating for 10 min, the protoscoleces treated with 50 μg/mL TPEDCC-PEG showed evaginated form, wrinkled and contracted soma region, and loss of hooks (Fig 4Bd). Treatment with 150 μg/mL TPEDCC-PEG led to significant collapse of protoscoleces, shedding of microtriches, rough and wrinkled surface, rostellar disorganization, and loss of hooks (Fig 4Be). After 250 μg/mL TPEDCC-PEG treatment, the protoscoleces showed complete disruption of the tegument with absence of microtriches, and severe distortion of suckers (Fig 4Bf).

**Fig 5:**
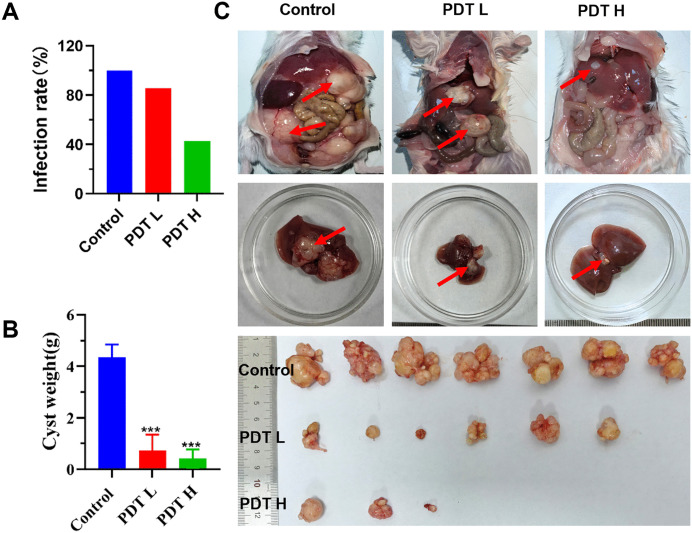
TPEDCC-PEG PDT effectively inhibits *E. multilocularis* protoscoleces. Infection rates (A) and cyst weights (B) 10 weeks after injection of TPEDCC-PEG pre-photodynamic treated protoscoleces (n = 2000) into BALB/c mice. (C) Gross morphology of metacestodes. PDT L, 12.5 μg/mL TPEDCC-PEG; PDT H, 150 μg/mL TPEDCC-PEG. ***p < 0.001 vs. control.

To further validate the protoscolicidal efficacy of TPEDCC-PEG PDT on protoscoleces, BALB/c mice were inoculated with protoscoleces that had been pre- photodynamic - treated with TPEDCC-PEG or untreated control *in vitro*. The animals were humanely euthanized 10 weeks post-infection to assess the parasite load. The findings indicated infection rates of 100%, 85.71%, and 42.85% in the control, 12.5 μg/mL TPEDCC-PEG, and 150 μg/mL TPEDCC-PEG group (Fig 5A), respectively. Notably, a significant decrease in the weight of metacestode cysts was observed in 12.5 μg/mL TPEDCC-PEG (0.73 ± 0.62 g) and 150 μg/mL TPEDCC-PEG (0.41 ± 0.35 g) groups, compared with the control group (4.34 ± 0.50 g) (Fig 5B). Upon examination of the metacestodes from each group, it was observed that the control mice exhibited a high prevalence and large-sized metacestodes in the liver, with extensive invasion of organs such as the intestines, kidneys, and spleen in abdominal cavity, characterized by numerous translucent vesicles. In contrast, the mice in 12.5 μg/mL TPEDCC-PEG group exhibited fewer and smaller metacestodes in liver, along with reduced metastasis to and invasion of other abdominal organs. In 150 μg/mL TPEDCC-PEG group, only one mouse (out of the 3 infected mice) exhibited minimal infection with metacestodes in liver, with no evidence of invasion by vesicular outgrowth into other organs (Fig 5C).

### Effects of TPEDCC-PEG PDT on ROS and GSH level and mitochondrial function of *E. multilocularis* protoscoleces

**Fig 6:**
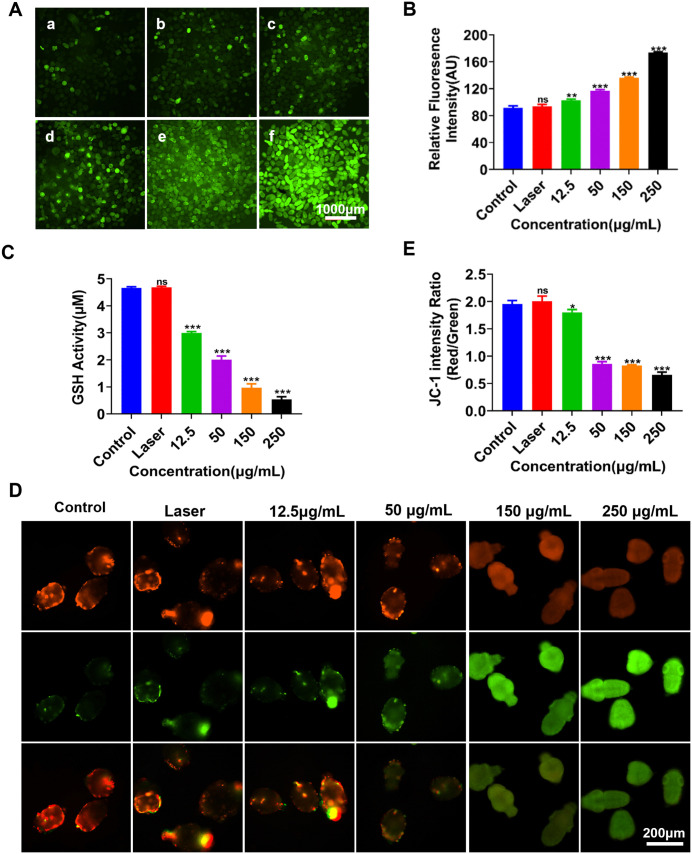
Detection of ROS and GSH levels and mitochondrial function of protoscoleces after laser irradiation. (A) ROS fluorescence images of protoscoleces incubated with TPEDCC-PEG with or without laser irradiation (410 nm, 10 min). (a) Control group. (b) Laser group. Protoscoleces irradiated by laser (410 nm, 10 min) after incubation with 12.5 μg/mL (c), 50 μg/mL (d), 150 μg/mL (e), and 250 μg/mL (f) of TPEDCC-PEG, respectively. The scale bar is 1000 μm. (B) ROS fluorescence intensities of protoscoleces in above different groups. (C) GSH activities change analysis of protoscoleces. (D) Fluorescence images of protoscoleces in above different groups. The protoscoleces were stained with dye JC-1. The scale bar is 200 μm. (E) The JC-1 fluorescence intensity ratio in above different groups. *p < 0.05*,* **p < 0.01, ***p < 0.001 vs. control.

To investigate the effect of TPEDCC-PEG PDT on ROS level, the cellular ROS generation in protoscoleces after treatment was measured using a ROS detection probe DCFH-DA. This probe could be oxidized into highly fluorescent green 2’,7’- dichlorofluorescein in the presence of ROS. As shown in Fig 6A, bright green fluorescence was detected in the protoscoleces treated with TPEDCC-PEG under laser irradiation. The analysis showed that the relative fluorescence intensity in protoscoleces treated with TPEDCC-PEG PDT was higher than that in control and laser groups (Fig 6B). Meanwhile, the fluorescence intensity gradually increased with the increase of TPEDCC-PEG concentration, indicating the enhanced ROS production from TPEDCC-PEG PDT performance (Fig 6B).

The effect of TPEDCC-PEG PDT on GSH level in protoscoleces after treatment was further measured (Fig 6C). After incubation with 12.5 μg/mL TPEDCC-PEG under laser irradiation, GSH in protoscoleces was significantly decreased compared to the control. Moreover, GSH levels decreased significantly and continuously with the increase in TPEDCC-PEG concentration.

To investigate the effect of TPEDCC-PEG PDT on mitochondrial function, the mitochondrial membrane potential (MMP) of protoscoleces was assessed using the cationic carbocyanine dye JC-1, an excellent green fluorescent probe for evaluating changes in MMP. The relative ratio of red to green fluorescence was utilized to evaluate the extent of mitochondrial depolarization and mitochondrial function. As shown in Fig 6D and 6E, the protoscoleces in control and only laser groups showed no significant fluorescence. In contrast, increased green fluorescence and decreased red fluorescence were detected in the protoscoleces treated with increased concentrations of TPEDCC-PEG under laser irradiation, indicating the mitochondria damages in protoscoleces.

### TPEDCC-PEG PDT induces apoptosis of *E. multilocularis* protoscoleces

**Fig 7:**
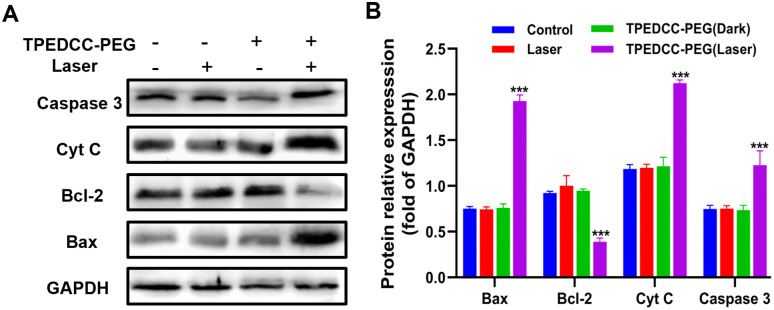
Anti-*E. multilocularis* mechanism of TPEDCC-PEG PDT. (A) and (B) show the expression of apoptosis-associated proteins Bcl-2, Bax, caspase-3, and Cyt C in protoscoleces as determined by western blot analysis. ***p *< *0.001 vs. control.

To investigate the effect of TPEDCC-PEG PDT on apoptosis of *E. multilocularis* protoscoleces, the key proteins involved in mitochondrial regulation of apoptosis were detected using western blot analysis. The expression of apoptosis-associated proteins, including Bcl-2, Bax, caspase-3, and Cyt C, was calculated (Fig 7A and 7B). Compared with other groups, the expression levels of caspase-3, Bax and Cyt C were significantly increased, while Bcl-2 was significantly decreased in the TPEDCC-PEG (laser) group.

### PDT exhibits therapeutic efficacy against *E. multilocularis* infection in mice

**Fig 8:**
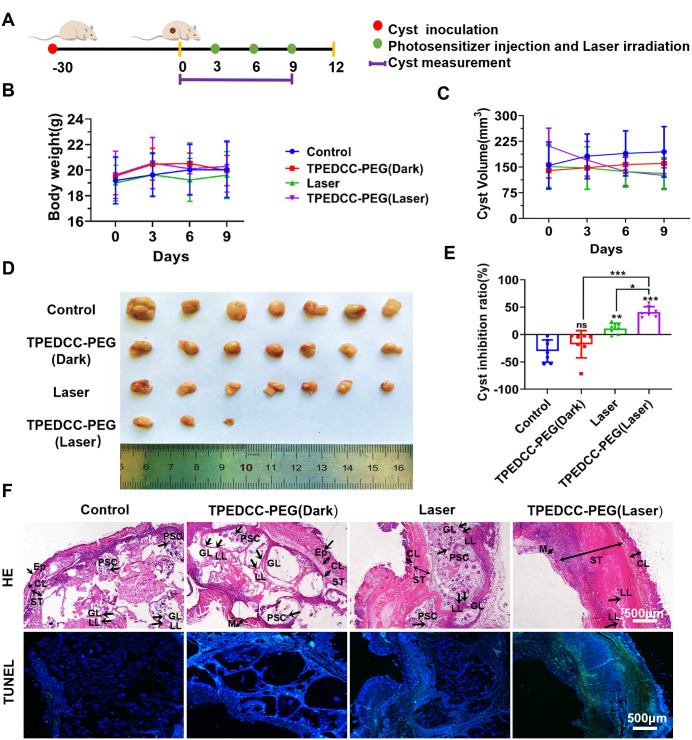
*In Vivo* PDT performance of TPEDCC-PEG in mice infected with *E. multilocularis* metacestode. (A) Mice were infected with 2000 *E. multilocularis* protoscoleces via subcutaneous inoculation. The metacestodes bearing mice were treated with laser irradiation (410 nm) for 10 min once every 3 days for a period of 9 days. (B) Body weight change after administration of PBS, TPEDCC-PEG (10 mg/kg, 50 μL) with or without laser irradiation (410 nm, 10 min). (C) Cyst volume change analysis of metacestodes bearing mice. (D) Gross morphology of metacestodes. (E) The analysis of cyst inhibition rate. (F) *E. multilocularis* cysts were observed by H&E and TUNEL staining. The scale bar is 500 μm. LL, laminated layer; GL, germinal layer; PSC, protoscoleces; Ep, epidermis; CL, corium layer; ST, subcutaneous tissue; M, muscle. *p < 0.05, **p < 0.01, ***p < 0.001.

To investigate the *in vivo* therapeutic effect of TPEDCC-PEG PDT against AE, BALB/c mice were infected with *E. multilocularis* protoscoleces via subcutaneous inoculation (Fig 8A). As shown in Fig 8C, after being treated with TPEDCC-PEG (10 mg/kg) every 3 days without laser irradiation, the volume of metacestode cysts in mice increased continuously and no abnormal changes in body weight were observed, which were similar to those in control mice (Fig 8B). The TPEDCC-PEG (Laser) group, that is, TPEDCC-PEG (10 mg/kg) with laser irradiation, resulted in a significant inhibition in growth of metacestodes by 45% (Fig 8C–8E) compared to control mice, laser treated mice, as well as TPEDCC-PEG (10 mg/kg) treated mice without laser irradiation. Remarkably, metacestodes in four mice completely disappeared after PDT with TPEDCC-PEG. Moreover, no death and adverse effects occurred in the treated mice during the experiment. The parasites from mice in control group and the TPEDCC-PEG without laser irradiation treated groups exhibited an intact typical structure of *E. multilocularis* metacestodes: the cysts were surrounded by the epidermal and dermal layer of the mice skin, with clear germinal and laminated layers, meanwhile, protoscoleces were still observed (Fig 8F). In contrast to other groups, the residual metacestodes from TPEDCC-PEG PDT mice exhibited severe damages, with almost complete lysis of the germinal layer, and the laminated layer displaying partial fragmentation and fragmented structures (Fig 8F). In addition, we performed TUNEL staining to analyze the effect of TPEDCC-PEG PDT on apoptosis of *E. multilocularis* metacestodes in mice (Fig 8F). The results demonstrated that the integral optical density values of FITC fluorescence in the metacestodes treated with TPEDCC-PEG PDT were significantly higher than those in the control, laser irradiation and TPEDCC-PEG alone–treated metacestodes.

**Fig 9:**
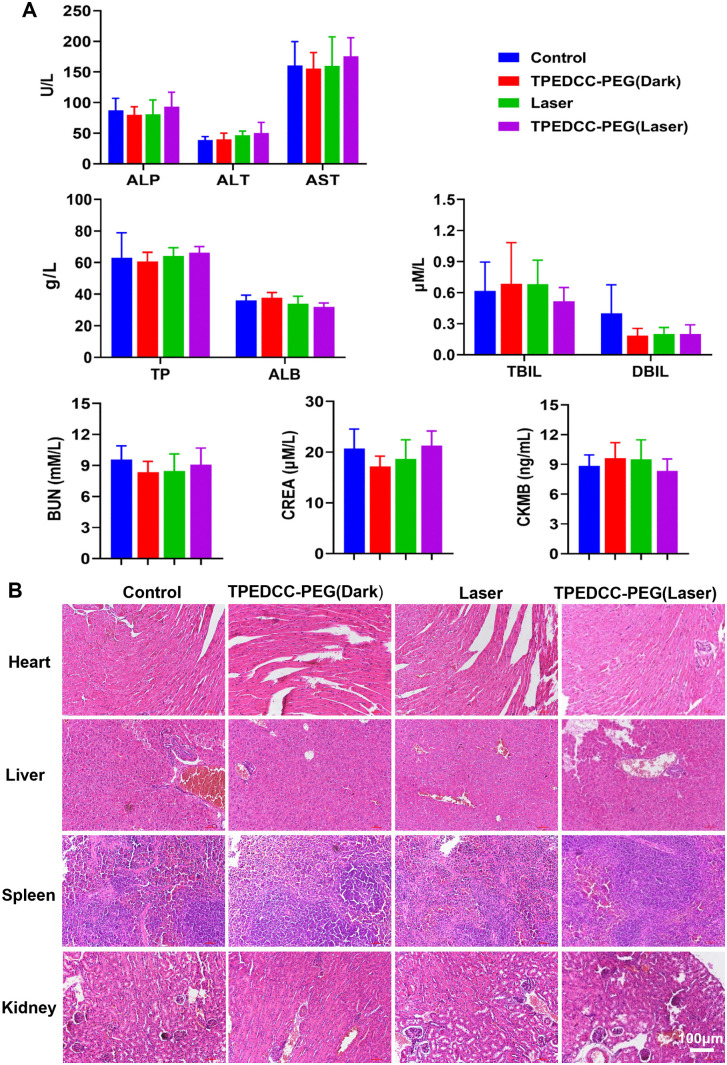
Serum biochemical analysis and histopathological examination of mice. (A) The serum biochemical parameters of mice after administration of PBS, TPEDCC-PEG (10 mg/kg, 50 μL) with or without laser irradiation (410 nm, 10 min). Total protein (TP), albumin (ALB), total bilirubin (TBIL), direct bilirubin (DBIL), alanine aminotransferase (ALT), aspartate aminotransferase (AST), alkaline phosphatase (ALP), urea nitrogen (BUN), creatinine (CREA), and creatine kinase isoenzyme MB fraction (CKMB) were detected. (B) Representative H&E staining images of the heart, liver, spleen, and kidney of mice. The scale bar is 100 μm.

Furthermore, the serum levels of biochemical parameters for liver, kidney, and heart function, total protein (TP), albumin (ALB), total bilirubin (TBIL), direct bilirubin (DBIL), alanine aminotransferase (ALT), aspartate aminotransferase (AST), alkaline phosphatase (ALP), urea nitrogen (BUN), creatinine (CREA), and creatine kinase isoenzyme MB fraction (CKMB), showed no significant changes after mice were treated with TPEDCC-PEG (Fig 9A). The further H&E staining of liver, kidney, heart and spleen was compatible with the results of biochemical parameters (Fig 9B).

## Discussion

AE is a life-threatening but neglected zoonotic disease that causes serious public health problems and economic losses, especially in developing countries [36]. The major treatment option of AE is surgical resection complemented by pre- and postoperative anti-parasitic treatment. However, the efficacy of surgical intervention is substantially mitigated by its contraindications and the high incidence of postoperative recurrence. Therefore, exploring novel therapeutic strategies for AE is crucial.

In recent years, PDT has been widely used in many medical fields, including cancer therapy, alleviation of autoimmune disease complications, wound healing improvement, and control or elimination of viral, fungal and bacterial infections in both planktonic and biofilm forms [25,37]. Furthermore, PDT has also shown notable anti-parasitic activity. A previous study demonstrated that PDT could induce the death of *Trichomonas vaginalis* and reduce its infection in animals [38]. Gong et al. also found that PDT combined with albendazole sulphoxide NPs could significantly inhibited the viability of *Echinococcosis granulosus* [39]. Therefore, in this study, we assessed the potential therapeutic implications of PDT against *E. multilocularis* through *in vitro* and *in vivo* experiments.

PS is the critical factor in determining the efficacy of PDT. However, the widely used traditional PSs have inevitable limitations, including aggregation under physiological conditions, aggregation-caused quenching effect, and reduced ROS generation, all of which compromise PDT efficacy [24,40]. To address these issues, a variety of nanophotosensitizers have been developed. Among them, newly developed PSs designed for higher ROS yield, particularly aggregation-induced emission (AIE) – based PSs have been proven the best candidates with enhanced ROS production [41]. Therefore, we developed a novel nanophotosensitizer TPEDCC-PEG through liposome encapsulation, using TPEDCC (an AIE PS with symmetric dicarboxylic acid groups) and DSPE-PEG as precursor materials. TPEDCC-PEG exhibits aggregation-induced red fluorescence emission. This property enables the visualization of cell death and greatly facilitates both disease diagnosis and treatment.

The size, optical properties, surface charge, and stability are pivotal factors that influence the enrichment of nanophotosensitizers at the lesion site and PDT efficacy [42–44]. In our investigation, the UV spectrum of TPEDCC-PEG (with a maximum absorption at 350 nm) is consistent with that of TPEDCC, indicating the successful loading of DSPE-PEG via liposome encapsulation (Fig 2C). Furthermore, the diameter of TPEDCC-PEG is approximately 100 nm, and it forms a monodisperse system with relatively uniform particle size (Fig 2A and 2B). This nano-sized structure would contribute to the passive targeting delivery of TPEDCC-PEG to the lesion site via the enhanced permeability and retention (EPR) effect, with prolonged circulation time in the blood [45]. As is known, the optimal surface charge for nanomedicines should be neutral or slightly negative. This is because several studies have demonstrated that nanomedicines with positive surface charge or cationic polymers can readily bind to the surface of vascular endothelial cells, thereby reducing drug accumulation. In contrast, the nanoparticles with highly negative charge can be easily captured by the reticuloendothelial system [45,46]. Thus, the slightly negative charge possessed by TPEDCC-PEG meets the ideal surface charge requirement of nanodrugs (Fig 2D). TPEDCC-PEG exhibited excellent colloidal stability in both aqueous solutions and DMEM medium supplemented with 10% FBS, ensuring prolonged stability during toxicological detection experiments (Fig 2E and 2F). Furthermore, subsequent assessment of cytotoxicity in different cells lines (Human Normal Liver LO2 cells, Human Dermal Fibroblasts cells, and Mouse Fibroblast L929 cells) indicates good biocompatibility of TPEDCC-PEG (Fig 2G–2I). Therefore, our results demonstrate that the novel nanophotosensitizer TPEDCC-PEG has excellent photodynamic properties and biosafety, and may be a promising photosensitizer.

*E. multilocularis* metacestodes display characteristics of asexual, unbounded, and infiltrative proliferation within human hosts. The germinal layer of metacestodes comprises densely packed tissue that contains undifferentiated stem cells, glycogen storage cells, connective and muscle tissue cells, as well as nerve cells [47]. The germinal layer undergoes budding towards the interior, giving rise to brood capsules, which subsequently produce protoscoleces, thereby highlighting its crucial role in the parasite’s survival strategy. Therefore, protoscoleces are usually used as an important model for studying *E. multilocularis* development and host-parasite interactions. To investigate the effect of TPEDCC-PEG nanoparticle-mediated PDT against *E. multilocularis*, an *in vitro* efficacy study was performed on *E. multilocularis* protoscoleces. Leveraging the inherent red fluorescence of TPEDCC-PEG, we assessed the vitality of protoscoleces after treatment with varying laser exposure times and TPEDCC-PEG concentrations. The results demonstrated that TPEDCC-PEG PDT exerted a significant lethal effect on protoscoleces *in vitro*, with the efficacy positively correlated with both irradiation duration and TPEDCC-PEG concentration (Fig 3). Notably, light exposure alone did not significantly alter the vitality of protoscoleces, suggesting that the thermal effect of light is not the predominant mechanism for protoscoleces destruction (Fig 3D). Inverted microscopy revealed that TPEDCC-PEG PDT induced eversion of protoscoleces, resulting in reduced volume, membrane defects, and a decrease in calcareous corpuscles (Fig 4A). Further SEM analysis confirmed the effects of TPEDCC-PEG against protoscoleces on the ultrastructural level, showing loss of hooks, shedding of microtriches, a rough and wrinkled surface, and disorganization of the rostellar structure (Fig 4B). These morphological and ultrastructural alterations were more severe with higher concentrations of TPEDCC-PEG PDT. Furthermore, the *in vivo* viability test (testing of viability by injection of tissue into rodents [48]) performed in BALB/c mice demonstrated significant decrease of infection rate and cysts weight in the mice infected with TPEDCC-PEG treated protoscoleces (Fig 5A and 5B), clearly confirming the potent protoscolicidal effect of TPEDCC-PEG PDT. Collectively, our *in vitro* efficacy study on protoscoleces demonstrated that TPEDCC-PEG PDT has a potent killing effect against protoscoleces in a short time.

ROS play pivotal roles in modulating myriad of physiological processes within organisms, their inherent biochemical attributes serving as the foundation for the essential mechanisms that govern growth, health maintenance, and senescence [49]. Numerous studies have reported that PDT performs cytotoxicity by utilizing the ROS generated upon laser irradiation in the tumor tissue [26,50,51]. To explore the underlying mechanisms of TPEDCC-PEG PDT against *E. multilocularis*, cellular ROS generation was assessed in protoscoleces after treatment. TPEDCC-PEG PDT induced an increase of ROS in protoscoleces in a dose-dependent manner (Fig 6A), indicating that the significantly enhanced ROS levels might contribute to its effectiveness against *E. multilocularis*. Moreover, the JC-1 staining assay indicated that TPEDCC-PEG PDT could induce membrane potential depolarization of protoscoleces and this effect was dose-dependent (Fig 6D and 6E). Most importantly, the JC-1 staining results were in good agreement with the ROS generation date. As is known, mitochondria are the most important organelle of cellular metabolic processors and primary targets for ROS [44]. Excessive generation of ROS will increase the degree of oxidation in cell. Once the degree of oxidation exceeds the antioxidant capacity of the cells for oxide scavenging, that is the imbalance between oxidation system and antioxidant system, the excess ROS could disrupt MMP [52], cause cellular dysfunction, protein and lipid peroxidation, DNA damage, and eventually lead to irreversible cell damage and death [53]. Furthermore, GSH is a key component of the cellular antioxidative system, essential for scavenging excessive ROS and detoxify xenobiotics [54]. Therefore, the cellular GSH levels was assessed to determine oxidative stress upon TPEDCC-PEG PDT. We observed that TPEDCC-PEG PDT suppressed GSH levels in protoscoleces in a dose-dependent manner (Fig 6C). Thus, these results suggest that TPEDCC-PEG PDT generates sufficient intracellular ROS to induce oxidative stress and mitochondrial damage in protoscoleces, leading to irreversible cellular damage.

**Fig 10:**
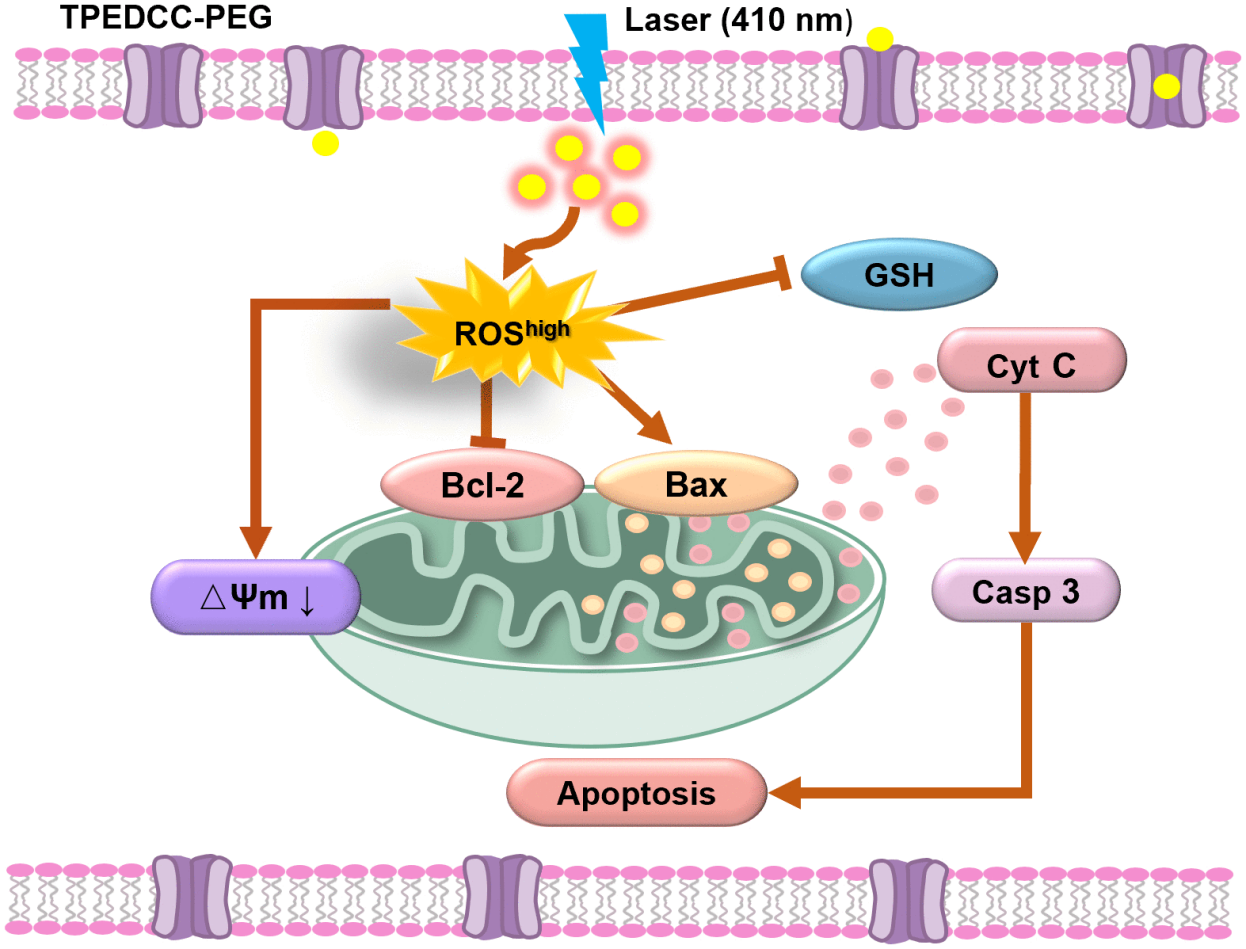
Schematic diagram of the anti-*E. multilocularis* mechanism of TPEDCC-PEG PDT.

The PDT induced cellular damage can cause cell death via various pathways, with apoptosis being the predominant mechanism [42,43]. It is important to note that there are significant distinctions among the apoptosis induced by PDT, radiotherapy, and chemical treatment. Previous studies have indicated that radiation and chemotherapeutic regimens predominantly induce apoptosis by damaging DNA, triggering cell-cycle checkpoints, growth arrest, and activation of the p53. In contrast, PDT primarily elicits an acute stress response involving mitochondrial damage, Cyt C release, and formation of an apotosome involving caspase [50]. Our results indicated that TPEDCC-PEG PDT can induce a significant increase of ROS and lead to mitochondrial impairment in protoscoleces (Fig 6D and 6E). Given the well-established link between ROS-mediated oxidative stress, mitochondrial dysfunction, and the onset of apoptosis [55], we hypothesized that apoptosis may be a crucial pathway for TPEDCC-PEG PDT leading to protoscoleces death. This hypothesis was supported by our observations in the protoscoleces after TPEDCC-PEG PDT, where the expression levels of caspase-3, Bax and Cyt C were significantly increased, while Bcl-2 was significantly decreased (Fig 7A and 7B). Based on these collective findings, we propose a working model (Fig 10) in which TPEDCC-PEG PDT down-regulate anti-apoptotic protein Bcl-2, up-regulate pro-apoptotic protein Bax, facilitate the release of Cyt C, and enhances caspase-3 expression, eventually activating the mitochondria-mediated apoptosis pathway of protoscoleces. It is important to note that while our study strongly associates the increase in ROS levels with apoptosis induction, the precise cause-effect relationship warrants further investigation using specific ROS scavengers in future studies.

To investigate the *in vivo* therapeutic effect of TPEDCC-PEG PDT, BALB/c mice were inoculated subcutaneously with *E. multilocularis* protoscoleces. The *in vitro* effect of TPEDCC-PEG PDT against *E. multilocularis* was subsequently confirmed in this mice model of AE. TPEDCC-PEG (10 mg/kg) PDT significantly inhibited the growth of *E. multilocularis* metacestodes in the infected mice compared with the untreated mice, achieving a 45% reduction in parasite volume (Fig 8E), thereby indicating the potent therapeutic efficacy of TPEDCC-PEG in the treatment of *E. multilocularis* infection. Moreover, it is important to note that the TPEDCC-PEG PDT was only performed three times and the duration of treatment was no more than 12 days, suggesting that TPEDCC-PEG PDT represents a significant and advantageous approach to enhancing treatment efficacy and reducing treatment duration in treating AE. The histological investigation further corroborated the efficacy of TPEDCC-PEG PDT in the treatment of AE (Fig 8F). The structural integrity of the *E. multilocularis* metacestodes was severely compromised after PDT treatment with TPEDCC-PEG, with the germinal layer detaching from the outer laminated layer, accompanied by substantial damage to germinal layer and fragmentation of the laminated layer. Notably, no protoscoleces were observed within the metacestodes treated with TPEDCC-PEG PDT. In contrast, the metacestodes from control mice, the laser irradiation mice, as well as the mice treated with TPEDCC-PEG without laser irradiation all displayed intact structures, with varying numbers of protoscoleces present in the cysts. Thus, the results indicated that the metacestodes treated with TPEDCC-PEG without laser irradiation did not experience significant damages, indicating the good biological safety of TPEDCC-PEG. Moreover, the metacestodes from the mice treated with TPEDCC-PEG PDT showed the significantly highest apoptosis rate, which might be responsible for the great effectiveness of TPEDCC-PEG PDT against *E. multilocularis* (Fig 8F)*.*

Furthermore, given its potent therapeutic efficacy, the *in vivo* biosafety of TPEDCC-PEG was evaluated. Throughout the treatment period, none of the mice exhibited adverse effects. Additionally, blood biochemical analysis (ALP, ALT, AST, ALB, TP, TBIL, DBIL, BUN, CREA, and CKMB) revealed no significant differences and no damage in normal organs after treatment with TPEDCC-PEG (Fig 9), indicating that TPEDCC-PEG administration did not compromise the organ function. Thus, TPEDCC-PEG demonstrated strong *in vivo* therapeutic efficacy against *E. multilocularis* while maintaining excellent biocompatibility and biosafety. However, since only a single infection of mice experiment was performed in the present study, more *in vivo* assay should be included in our further study to validate and support the potential of TPEDCC-PEG for future clinical application in the treatment of human AE.

In summary, in the present study, we successfully developed a nanophotosensitizer TPEDCC-PEG, and investigated its PDT therapeutic effect against *E. multilocularis* infection for the first time. TPEDCC-PEG exhibited outstanding features as a photosensitizer for AE treatment, including the nano-sized structure, highly negative charge, excellent optical property, colloidal stability, and biocompatibility. Treatment with TPEDCC-PEG combined with laser irradiation exhibits highly effective *in vitro* effect against *E. multilocularis* protoscoleces. The *in vivo* results also consolidate the superior anti-AE therapeutic effect of TPEDCC-PEG PDT. Furthermore, TPEDCC-PEG PDT can generate intracellular ROS and decrease GSH levels. The anti-*E. multilocularis* mechanism of TPEDCC-PEG PDT is associated with the activation of the mitochondria-mediated apoptosis pathway, characterized by the disruption of mitochondrial membrane potential and altered expression of apoptosis-associated proteins. Taken together, our study demonstrates that the PDT treatment of photosensitizer TPEDCC-PEG, which exhibits a profound effect against *E. multilocularis* with no host toxicity both *in vitro* and *in vivo*, is a promising strategy for AE therapy.
